# Marine Nucleosides: Structure, Bioactivity, Synthesis and Biosynthesis

**DOI:** 10.3390/md12125817

**Published:** 2014-12-02

**Authors:** Ri-Ming Huang, Yin-Ning Chen, Ziyu Zeng, Cheng-Hai Gao, Xiangdong Su, Yan Peng

**Affiliations:** 1 Key Laboratory of Plant Resources Conservation and Sustainable Utilization, South China Botanical Garden, Chinese Academy of Sciences, Guangzhou 510650, China; E-Mail: huangriming@scib.ac.cn; 2 Department of Pharmacy and Pharmacology, University of Bath, Bath BA2 7AY, UK; E-Mail: prsxs@bath.ac.uk; 3 College of Light Industry and Food Engineering, Guangxi University, Nanning 530004, China; E-Mail: chendianyu3356@163.com; 4 School of Pharmaceutical Sciences, Shandong University, Jinan 250012, China; E-Mail: zzy205687@126.com; 5 Key Laboratory of Marine Environmental Science, Guangxi Academy of Sciences, Nanning 530007, China; E-Mail: gaochenghai@gxas.cn; 6 Life Science & Technology School, Lingnan Normal University, Zhanjiang 52048, China

**Keywords:** biological activity, biosynthetic pathway, marine organism, nucleoside, synthesis

## Abstract

Nucleosides are glycosylamines that structurally form part of nucleotide molecules, the building block of DNA and RNA. Both nucleosides and nucleotides are vital components of all living cells and involved in several key biological processes. Some of these nucleosides have been obtained from a variety of marine resources. Because of the biological importance of these compounds, this review covers 68 marine originated nucleosides and their synthetic analogs published up to June 2014. The review will focus on the structures, bioactivities, synthesis and biosynthetic processes of these compounds.

## 1. Introduction

**Figure 1:**
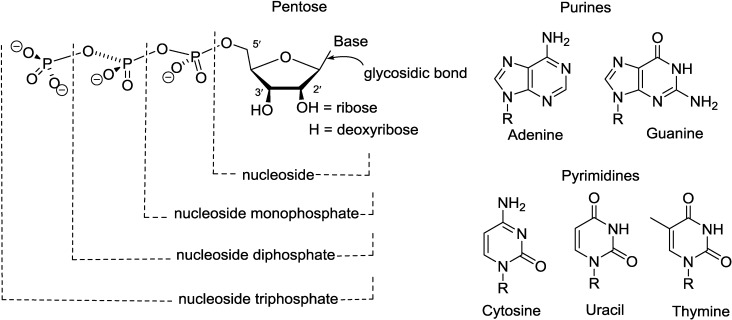
The structural compositions of nucleosides and nucleotides.

Nucleosides belong to a class of organic compounds with their structures being composed of a nitrogen-containing heterocyclic nucleobase and a 5-carbon sugar (Figure 1). The nucleobase is bound to the 5-carbon sugar, either a ribose or a deoxyribose, through a β-glycosidic linkage. Phosphorylation of nucleosides on the primary hydroxyl group of the sugar moiety forms nucleotides, the building blocks of DNA and RNA. Both nucleosides and nucleotides are vital components of all living cells and involved in several fundamental biological processes. Discoveries made in the area of purine and pyrimidine nucleosides and nucleotide chemistry [1,2,3] have contributed substantially to the better understanding of the biological processes at the molecular levels. Therefore scientists show great interest in not only the naturally occurring nucleosides and their biochemical properties but also the effects of synthetic nucleosides on living organisms [4,5,6].

Microorganisms and marine organisms are capable of producing different nucleosides with unusual structures and biological properties [7,8,9]. Some of these molecules with significant bioactivities have been isolated previously from a variety of marine resources [8,9]. The discoveries of these remarkable biological activities in the marine nucleosides have promoted a great amount of research work on the synthesis of various analogs of these nucleosides and the further evaluation of their biological activities [10,11,12]. The searching for novel analogs of natural nucleosides with potential antibiotic, antiviral, antiparasitic and antitumor properties has driven the rapid progress in the area of nucleoside chemistry research [13].

Bioactive marine nucleosides have been reviewed [14,15]. However, there is no comprehensive review of marine nucleosides since the first unusual marine nucleoside was isolated by Bergmann in 1950 [14]. Thus, this review aims to summarize 68 marine-derived nucleosides and their synthetic analogues reported up to the first half year of 2014. The structures, bioactivities, synthesis and biosynthetic processes of these marine derived nucleosides are included.

## 2. Purine Nucleosides

**Figure 2:**
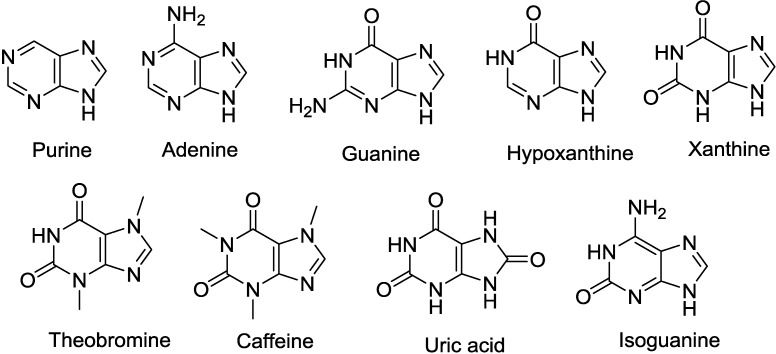
The structures of the common purine derivatives.

Purine nucleoside is composed of a purine base and a five-carbon sugar (either a ribose or a deoxyribose). The common purine bases include purine, adenine, guanine, hypoxanthine, xanthine, theobromine, caffeine, uric acid and isoguanine (Figure 2).

### 2.1. Purine-d-Arabinosides

**Figure 3:**
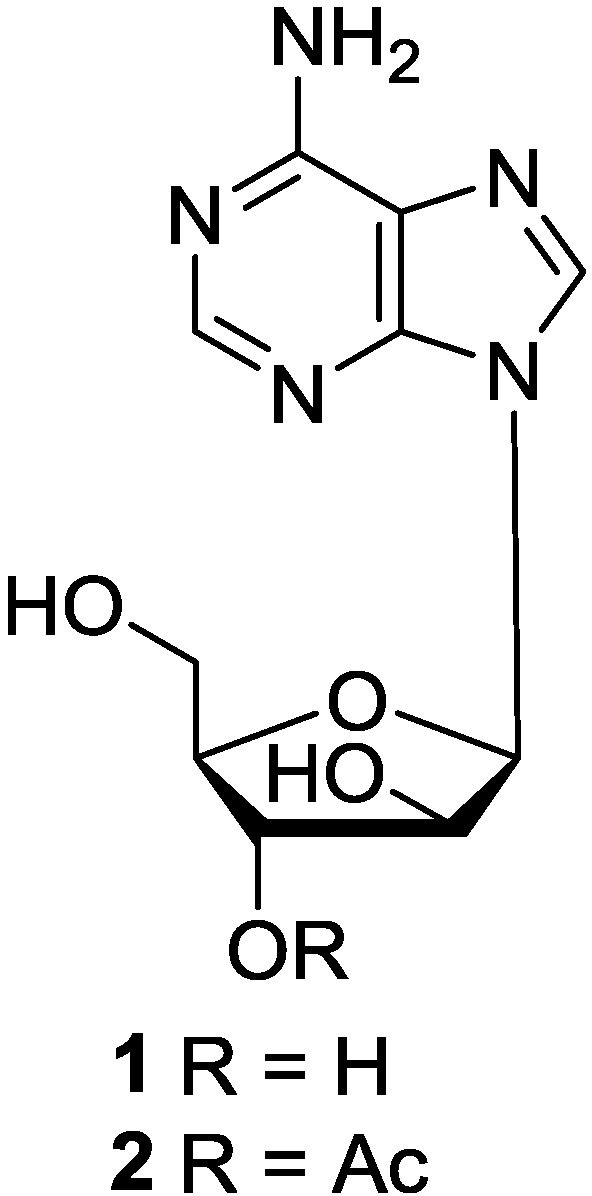
The structures of purine-d-arabinosides (**1** and **2**).

Spongoadenosine (Ara-A, **1**) and its 3′-*O*-acetyl derivative (**2**) were isolated from gorgonian *Eunicella cavolini* [16]. It is interesting to note that **1** was synthesized [17] prior to its isolation from a natural source. Ara-A exhibited significant antiviral activity against DNA viruses, which was the first antiviral drug for the treatment of fatal *Herpes encephalitis* [18]. The significance of its biological properties [11] has ensured continued interest since the compound was first synthesized in 1960 [19,20]. It was found that 9-β-d-darabinofuranosyl-adenine-5′-phosphate, which penetrated the cell without degradation had more sustained toxicity against mouse fibroblasts (L-cells) than that of its nucleoside. Ara-A had a firmly established role in the management of certain human herpes virus infections and was also effective in the therapy of *H. keratitis*, *H. encephalitis* and *Varicella zoster* infections in immunosuppressed patients [21]. In addition, the biosynthesis of Ara-A has been studied [22]. It was suggested that Ara-A is produced by direct epimerization of C-2′ hydroxy group of adenosine or a derivative thereof. It is also likely that a 2′-keto compound is involved as an intermediate [23]. However, there is no detailed report on biological activity of compound **2**. Since compound **2** has a very similar structure to compound **1**, it will be worthy to further investigate whether **2** has similar biological properties as **1**, or whether 2′-OH in **1** and 2′-AcO in **2** have their own contribution to the biological activity. In addition, as both compounds **1** and **2** were produced by the same gorgonian *E. cavolini*, they may have the same biosynthesis pathway in the gorgonian. The corresponding chemical structures are shown in Figure 3.

### 2.2. Purine-2′-Deoxyribosides 

**Figure 4:**
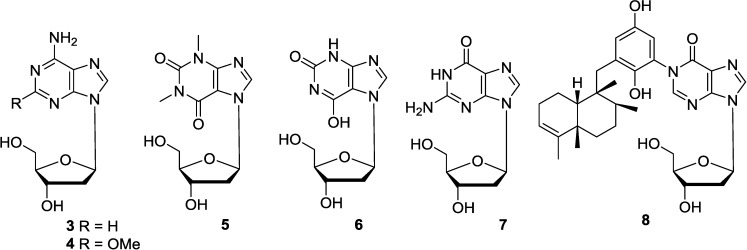
The structures of purine-2′-deoxyribosides (**3**–**8**).

2′-Deoxyadenosine (**3**), possessing significant biological properties [24], is a normal component of nucleic acids. However, it was isolated in free state from the marine sponge *Dasychalina cyathina* [25], and also isolated later from the marine sponge *Callyspongia* species [26]. 2′-Deoxy-spongosine (**4**), known as a synthetic product, was first isolated from the Caribbean sponge *Cryptotethia crypta* [27], and also isolated from the same sponge collected from Western Australia [28]. Aplysidine (**5**) was isolated from the Okinawan marine sponge *Aplysina* sp. Its structure was elucidated by spectroscopic methods and confirmed by the synthesis of aplysidine (**5**) [29]. This compound is the first nucleoside with theophylline ring as a base isolated from a marine source. Aplysidine (**5**) was found to have the antagonistic activity for adenosine A1 receptor comparable with xanthine-*N*-7-ribosides [30]. Xanthosine (**6**) was isolated from a starfish *Asterias rollestoni* (Yellow Sea, China) [31]. 2′-Deoxy-guanosine (**7**) was isolated from the marine sponge *Haliclona* sp. [32]. Avinosol (**8**) was isolated from the marine sponge *Dysidea* sp. collected in Papua New Guinea. Avinosol (**8**) was apparently the first example of a naturally occurring meroterpenoid-nucleoside conjugate, showing anti-invasion activity in a cell-based assay [33]. Among these purine-2′-deoxyribosides, seven of total eight compounds were isolated from marine sponges. Therefore, it is possible that this type compounds are mainly produced by marine sponge origin as they there share the similar biosynthesis pathways for purine-2′-deoxyribosides in marine sponges. The corresponding chemical structures are shown in Figure 4.

### 2.3. Purine-1-β-d-Ribosides

**Figure 5:**
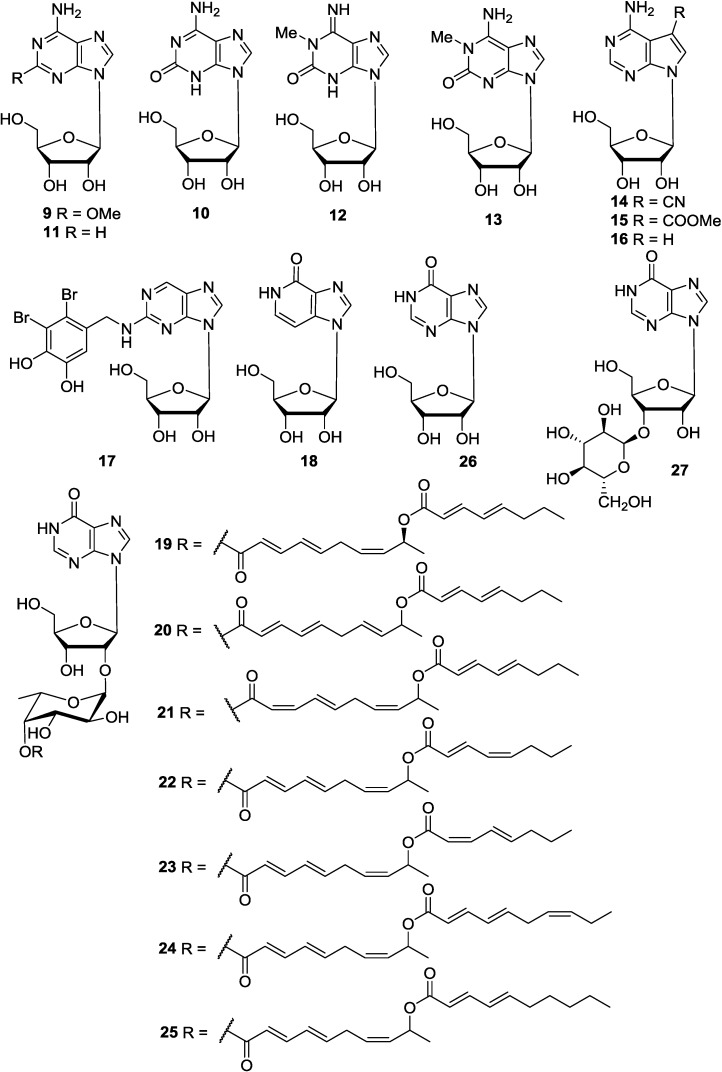
The structures of purine-1-β-d-ribosides (**9**–**27**).

Spongosine (**9**) was first isolated from the marine sponge *Cryptotethia crypta* in 1950 [14,34]. It was also obtained from the marine sponge *Haliclona* sp. [32] and the marine bryozoan *Bugula neritina* [35]. The structure of spongosine (**9**) as 3-methoxy adenosine, was confirmed by its chemical synthesis from 2-chloroadenine [36]. Three syntheses of spongosine (**9**) were reported [37,38]. Spongosine (**9**) was also synthesized from isoguanosine (**10**) through a new method [39]. Isoguanosine (**10**) was isolated from the marine nudibranch mollusk *Diaulula sandiegensis* (Monterey, California) [40]. Isoguanosine (**10**) exhibited hypotension, bradycardia and relaxation of smooth muscle activities [41]. The compound was more potent and much longer acting than adenosine. Like adenosine and its analogs, isoguanosine (**10**) stimulated accumulation of adenosine 3′,5′-monophosphate (cAMP) in brain tissue [41]. Effect of the size of alkoxy groups at position 2 in spongosine has been extensively studied with respect to its coronary vasodilating activity [42]. The result revealed that using an *n*-propoxy group at position 2 to replace a methoxy group resulted in eight-fold increase in its activity. Adenosine (**11**) was reported to be the cardioactive constituents of *D. cyathina* [25]. Its transportation, formation and inactivation in different tissues and involvement in the pathophysiology of renal changes observed in various types of renal insufficiency have been discussed [43]. The bronchodilation efficiency of methylxanthine is believed to be due to the adenosine antagonism action. The role of its uptake inhibitors as probe has been established [44]. Doridosine (**12**) [45,46,47] was isolated from marine sponge *Tedania digitata* and nudibranch *Anisodoris nobilis* at approximately the same time by two groups working independently in different continents (Australia and USA) [48,49]. The function and origin of this compound in these marine organisms is not known. Doridosine (**12**) exhibited muscle relaxant, hypothermic and cardiovascular effects following oral administration in mice and rats [46,47]. It interacted directly with adenosine receptors in guinea-pig brain to stimulate adenylate cyclase [50]. Its muscle relaxant activity and other properties have been compared with its various analogs. It has been found that the potency is retained in compounds in which the 1-methylisoguanine moiety is unaltered. 1-Methylisoguanosine (**13**) is a close derivative of doridosine (**12**) that occurs in the marine animals, which was isolated from the sponge *T. digitata* [45,51]. It has also been reported to occur in the nudibranch *A. nobilis* [48] and coral *Madracis mirabilis* [52]. This nucleoside showed potent muscle relaxant, blood pressure lowering, cardiovascular and anti-inflammatory activity [53,54]. Two known cytotoxic nucleosides, toyomycin (**14**) [55] and 5-(methoxycarbonyl)-tubercidin (**15**) [56], were isolated from *Jaspis johnstoni* from Fiji [57]. Tubercidin (**16**), a known nucleoside antibiotic, was isolated as the major metabolite from a marine sponge *Caulospongia biflabellata*. Tubercidin (**16**) exhibited potent cytotoxic activity against P388 and A549 tumor cells. The isolation, structure elucidation and biological activities of tubercidin (**16**) were described in the literature, which was the first report of the isolation of tubercidin from a marine organism [58]. A bromophenol coupled with deoxyguanosine (**17**) was isolated from *Rhodomela confervoides* (Qingdao, China) [59]. 3-Deazainosine (**18**), previously known as a synthetic compound [60], was isolated from the Egyptian ascidian *Eudistoma laysani* [61]. Shimofuridin A (**19**), a cytotoxic nucleoside derivative embracing an acylfucopyranoside unit, was isolated from the Okinawan marine tunicate *Aplidium multiplicatum* [62]. The compound has also been synthesized [63]. Shimofuridins B–G (**20**–**25**) with acylfucopyranoside moiety were minor metabolites from the same organism [64]. Inosine (**26**) was isolated from the extract of the didemnid ascidian *Lissoclinum* sp. [65]. The crustacean *Ligia exotica* from Korea contained 3′-*O*-(α-d-glucosyl)inosine (**27**), for which the structure was confirmed by a total synthesis [66]. Among these purine-1-β-d-ribosides, compounds **14**–**16** are cytotoxic metabolites. Further investigation on their pharmacological action mechanism and structure-activity-relationships (SAR) are needed to explore potential use for these compounds. In addition, shimofuridins B–G (**20**–**25**) represent compounds with interesting structural backbone. However, there is no reported biological data for this type of molecules. Their synthetic analogues are needed to further evaluate their biological properties. The corresponding chemical structures are shown in Figure 5.

### 2.4. Purine Nucleoside Analogues

**Figure 6:**
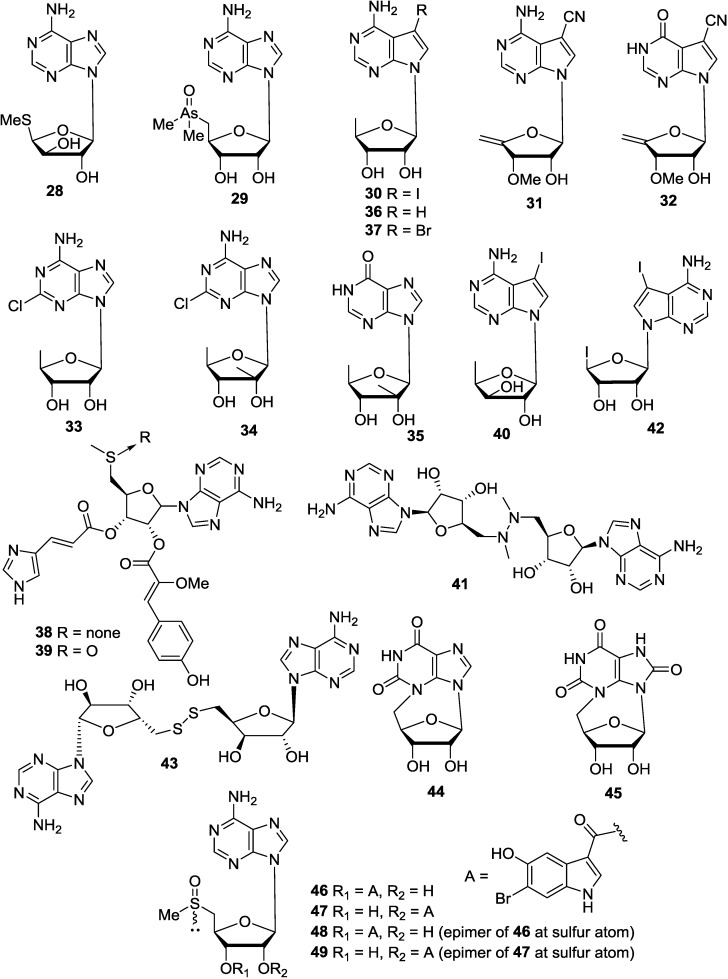
The structures of purine nucleoside analogues (**28**–**49**).

An unusual nucleoside isolated from marine nudibranch mollusk *Doris verrucosa* [67] was characterized as 9-[5′-deoxy-5′-(methylthio)-β-d-xylofuranosyl]adenine (**28**). It was the first naturally occurring analog of methylthio-adenosine (MTA). In the biological system MTA is formed from *S*-sadenosyl-l-methionine (AdoMet), a ubiquitous enzyme that occurs both in normal and malignant tissues. AdoMet acts as methyl group donor in transmethylation reaction. The nucleoside (**28**) was the first naturally occurring purine nucleoside carrying a substituted xylose sugar moiety. It has been synthesized [68] prior to its isolation from a marine nudibranch. An arsenic containing the nucleoside characterized as 5′-deoxy-5′-dimethylarsinyladenosine (**29**) was isolated from the kidney of the giant clam *Tridacna maxima* [69]. Marine alga *Hypnea valendia* [70] has furnished 5-iodo-5-deoxytubercidine (**30**), whose sugar moiety is 5-deoxyribose. The nucleoside (**30**) displayed prominent muscle relaxant property. It also produced hypothermia in mice [70]. A species of *Mycale* collected in Japan contained two novel nucleosides, mycalsines A (**31**) and B (**32**), both inhibited cell division in the fertilized-starfish-egg assay [71]. Kumusine (**33**) isolated from an Indonesian sponge *Theonella* sp. was characterized as 2-chloro-9-(5′-deoxy-2′-methyl-β-d-ribofuranosyl)-adenine [72]. 2′-*C*-methyl-5′-deoxyribofuranosyl nucleosides, trachycladine A (**34**) and trachycladine B (**35**), were isolated from the marine sponge *Trachycladus laevispirulifer* (Western Australia) [73]. The nucleosides 5′-deoxytubercidin (**36**) and 5′-deoxy-3-bromotubercidin (**37**) were isolated from *Didemnum voeltzkowi* from Philippines [74]. Australian *Atriolum robustum* was the source of amino acid-derived metabolites **38** and **39** [75]. Compound **38** was a relatively potent partial agonist of human A3 adenosine receptors. As an unusual nucleoside, 4-amino-7-(5′-deoxy-β-d-xylofuranosyl)-5-iodopyrrolo[2,3-*d*]pyrimidine (**40**), was isolated from an ascidian, *Diplosoma* sp. Compound **40** was found to inhibit the division of fertilized sea urchin eggs [76]. Ostrerine A (**41**) was isolated from extracts of the mollusk *Ostrea rivularis*, which is a food source and traditional Chinese medicine [77]. The iodinated nucleoside (**42**), isolated from *Diplosoma* sp. (Hateruma Is., Okinawa), inhibited the division of fertilized sea urchin eggs [76]. The first naturally occurring nucleoside disulfide (**43**) has been reported to be isolated from a dredges South Australian *T. laevispirulifer* [78]. A species of *Erylus* (Australia) was the source of *N^3^*,5-cycloxanthosine (**44**), the first naturally occurring cyclonucleoside. The compound is a known synthetic product prior to the isolation [79]. A new cyclonucleoside (**45**) was isolated from *Axinella polypoides* (Calvi, Corsica, France) [80]. Investigation of the secondary metabolites of the ascidian *Herdmania momus* led to the isolation and characterization of four new nucleoside derivatives (**46**–**49**) [81]. Structural studies showed that these derivatives could represent a series of rare methylsulfinyladenosine derivatives of interconvertible transesterification isomers and/or sulfinyl epimers. The antiviral activities of **46**–**49** were evaluated against a series of human pathogenic viruses [81]. The corresponding chemical structures are shown in Figure 6.

## 3. Pyrimidine Nucleosides

A pyrimidine nucleoside simply consists of a pyrimidine base and a five-carbon sugar (either a ribose or a deoxyribose). The pyrimidine base belongs to the diazines family with two nitrogen atoms position at 1 and 3 of the six-membered aromatic heterocyclic ring. In nucleic acids, pyrimidine nucleosides and pyrimidine nucleotides are composed of three types of nucleobases, they are cytosine (C), uracil (U) and thymine (T) (Figure 1).

### 3.1. Pyrimidine-d-Arabinosides

**Figure 7:**
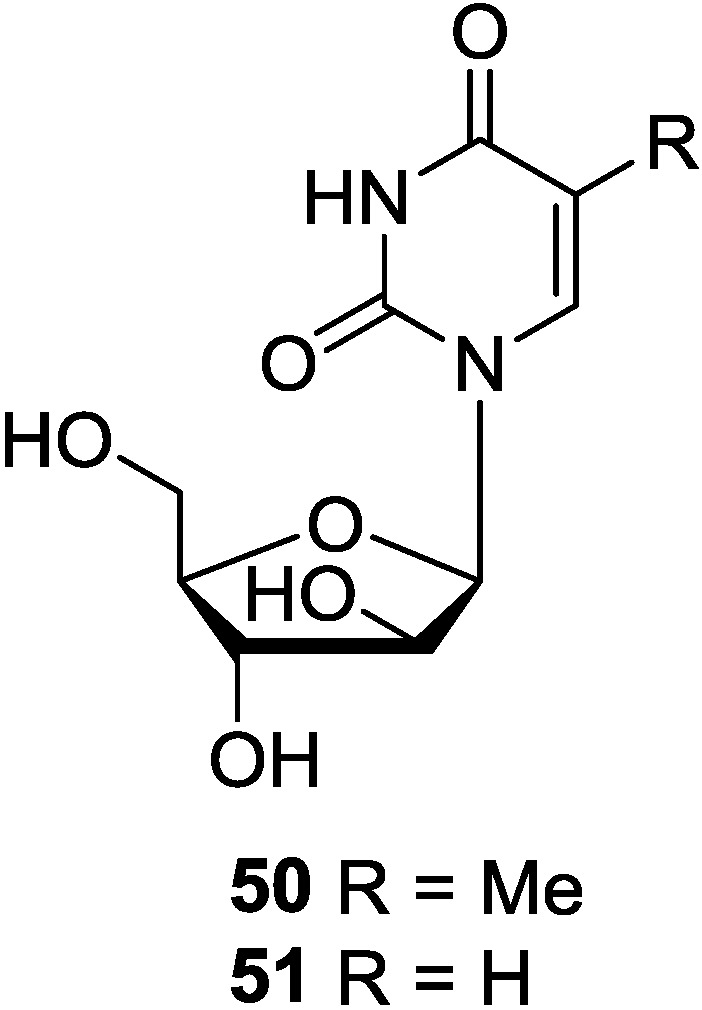
The structures of pyrimidine-d-arabinosides (**50** and **51**).

In 1950, Bergmann for the first time isolated an unusual nucleoside, spongothymidine (Ara-T, **50**) from the sponge *C. crypta* [14]. This pioneering work of late Prof. Bergmann stimulated a wide interest in the sponges as a source of novel compounds. Spongothymidine (**50**) was obtained from the sponge *C. crypta* by acetone extraction [34], and also isolated from the marine sponge *Callyspongia* sp. [26]. Spongothymidine (**50**) was found to be effective against HSV-l, HSV-2 and *V. zoster* virus (VSV) (ID_50_ 0.25–0.5 μg/mL) [82,83]. Its inhibition against HSV-l and HSV-2 was selective and was effective orally [82]. Ara-T was also effective against EMV, but inactive against CMV [83]. Extensive purification of the mixture of nucleosides obtained by Soxhlet extraction of the sponge *C. crypta* with acetone yielded spongouridine (**51**) [84]. Spongouridine (**51**) isolated first from a marine sponge, was subsequently obtained from the gorgonian *E. cavolini* [85]. Spongouridine has been used as a starting material for the synthesis of marine nucleoside, spongoadenosine (Ara-A), by a combination of chemical and microbial process [85]. Spongouridine was cleaved reversibly [86] to d-arabinose-l-phosphate and uracil by the enzyme nucleoside phosphorylase. Its phosphate has been prepared for antiviral evaluation. It showed weak antiviral properties [87] and very weak activity against HSV-l as compared to spongothymidine. The corresponding chemical structures are shown in Figure 7.

### 3.2. Pyrimidine-2′-Deoxyribosides 

**Figure 8:**
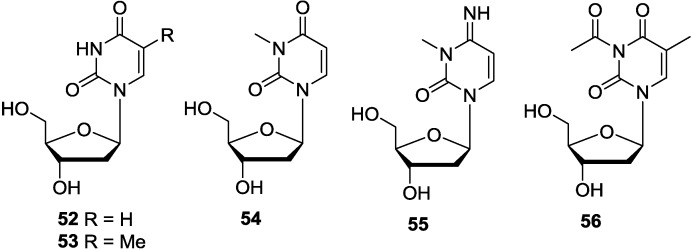
The structures of pyrimidine-2′-deoxyribosides (**52**–**56**).

1-(2′-Deoxy-β-d-ribofuranosyl) uracil (2′-deoxyuridine, **52**) and 1-(2′-deoxy-β-d-ribofuranosyl) thymine (thymidine, **53**) were isolated from starfish *Acanthaster planci* [88] and also isolated from the marine sponges *Haliclona* sp. [32] and *Callyspongia* sp. [26]. The usual pyrimidine nucleosides, 3-methyl-2′-deoxyuridine (**54**) and 3-methyl-2′-deoxycytidine (**55**) were identified as the metabolites of *Geodia baretti* that caused strong contractile activity in the guinea-pig ileum assay [89]. One new nucleoside derivative, named 3-acetyl-5-methyl-2′-deoxyuridine (**56**), was isolated from the cultures of *Streptomyces microflavus*. This strain was an associated actinomycete isolated from the marine sponge *Hymeniacidon perlevis* collected from the coast of Dalian (China) [90]. Based on the analysis of the origin of these pyrimidine-2′-deoxyribosides, they were produced by different species, such as starfish, marine sponges and symbiotic microorganisms. We therefore think whether these different species have some common gene clusters for producing this type of nucleosides are still needed more investigations. The corresponding chemical structures are shown in Figure 8.

### 3.3. Pyrimidine-1-β-d-Ribosides

**Figure 9:**
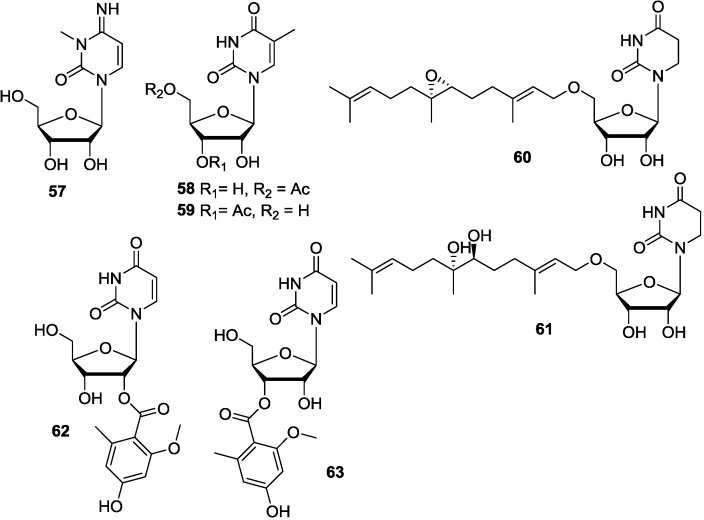
The structures of pyrimidine-1-β-d-ribosides (**57**–**63**).

3-Methylcytidine (**57**) is the only pyrimidine riboside isolated from marine sponge *G. baretti* [89], and **57** displayed contractile activity in the ileum assay. Thymidine analogues (**58**) and (**59**), previously known as synthetic compounds, were isolated from extracts of *Cladiella australis* (Taiwan) [91]. Mild cytotoxicity was exhibited by **58** and **59**. Famesides A and B (**60** and **61**), linear sesquiterpenoids connected by ether links to a ribose dihydrouracil nucleoside, were isolated from a marine-derived *Streptomyces* sp., strain CNT-372, grown in saline liquid culture. The farnesides are only the second example of this exceedingly rare class of microbial terpenoid nucleoside metabolites. Farneside A (**60**) was found to have modest antimalarial activity against the parasite *Plasmodium falciparum* [92]. Kipukasins H and I (**62** and **63**) were isolated from the fungus *Aspergillus versicolor* derived from the gorgonian *Dichotella gemmacea* collected in the South China Sea. Compounds **62** and **63** exhibited selective antibacterial activity against *Staphylococcus epidermidis* with a MIC value of 12.5 μM. This is the first report about their isolation, structure elucidation and biological activities of compounds **62** and **63** [93]. Apart from compound **60**, all these pyrimidine-1-β-d-ribosides in this article were found to possess interesting biological properties, further investigations on their pharmacological action mechanism should be carried out, and more analogs should be synthesized to understand their SAR. The corresponding chemical structures are shown in Figure 9.

### 3.4. Analogues of Pyrimidine Nucleosides

**Figure 10:**
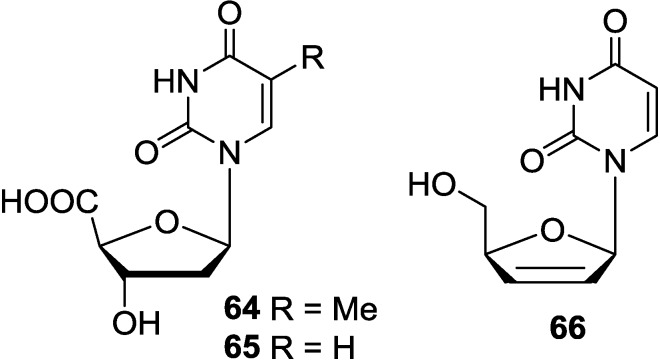
The structures of analogues of pyrimidine nucleosides (**64**–**66**).

Two unusual 2′-deoxy-nucleoside uronic acids, thymidine-5′-carboxylic acid (**64**) and 2′-deoxyuridine-5′-carboxylic acid (**65**), were isolated from the Mediterranean tunicate *Aplidium fuscum* [94]. The synthetically known antiviral agent 2′,3′-didehydro-2′,3′-dideoxyuridine (**66**) has been obtained from Okinawan marine sponge *Aplysina* sp. which was collected off the Kerama Islands, Okinawa [29]. The corresponding chemical structures are shown in Figure 10.

## 4. Indole Nucleosides

**Figure 11:**
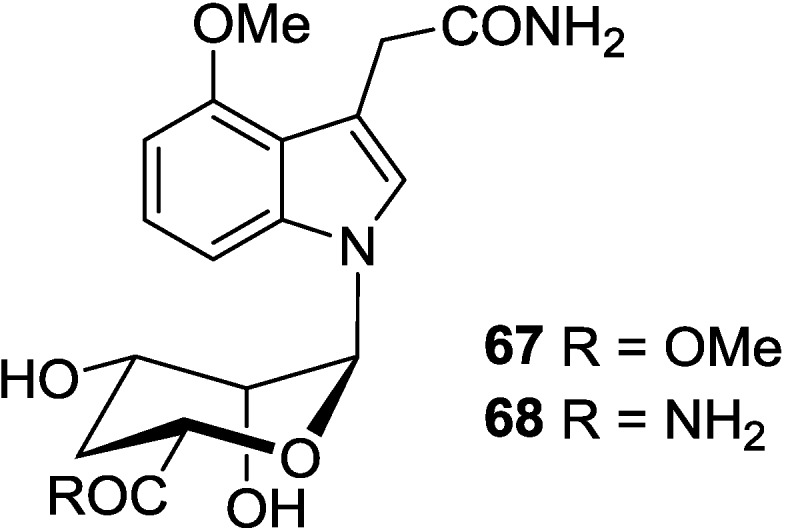
The structures of indole nucleosides (**67** and **68**).

The kahakamides A (**67**) and B (**68**) are two new indole nucleosides, which were isolated from the actinomycete *Nocardiopsis dassonvillei* obtained from a shallow water sediment sample from Kauai, Hawaii. Kahakamide A (**67**) exhibited slight inhibition of the Gram-positive bacterium *Bacillus subtilis* in a disc-diffusion assay [95]. These indole nucleosides are seldom found in marine organisms, it is necessary to clarify their biosynthesis pathways in natural resources, and to discover their biological functions. The corresponding chemical structures are shown in Figure 11.

## 5. Synthesis

Although studies for the synthesis of the nucleosides began in 1948 [96], the preparations of the nucleosides and their analogues are still a particularly challenging and attractive target for the synthetic community because of their promising pharmacological profiles.

**Scheme 1:**
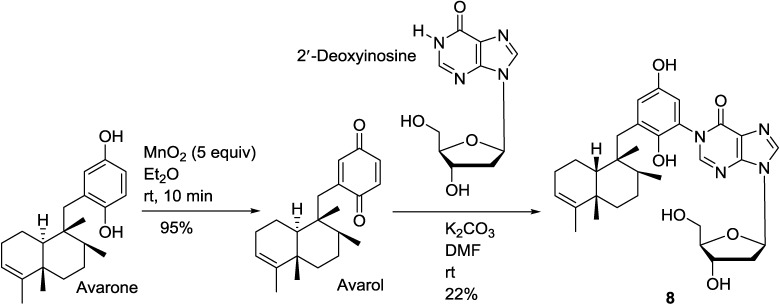
Synthesis of avinosol (**8**) from avarol.

To confirm the proposed structure of avinosol (**8**) [33], the natural product was synthesized from avarone and 2′-deoxyinosine as shown in Scheme 1. Avarone was prepared in quantitative yield by oxidation of naturally occurring avarol [97], obtained from the *Dysidea* sp. extract, with MnO_2_ in Et_2_O at room temperature for 10 min. Reaction of avarone with 2′-deoxyinosine in DMF and K_2_CO_3_ at room temperature for 30 min gave avinosol (**8**) in 22% yield (Scheme 1) [98].

**Scheme 2:**
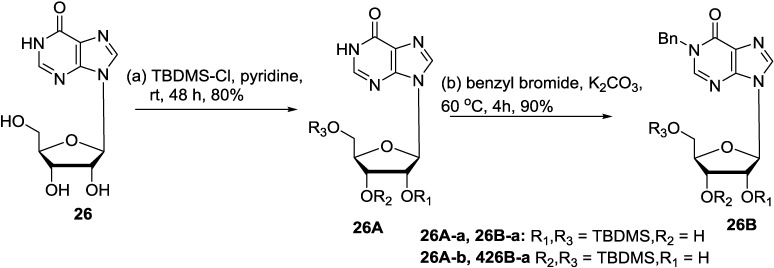
Synthesis from inosine (**26**).

**Scheme 3:**
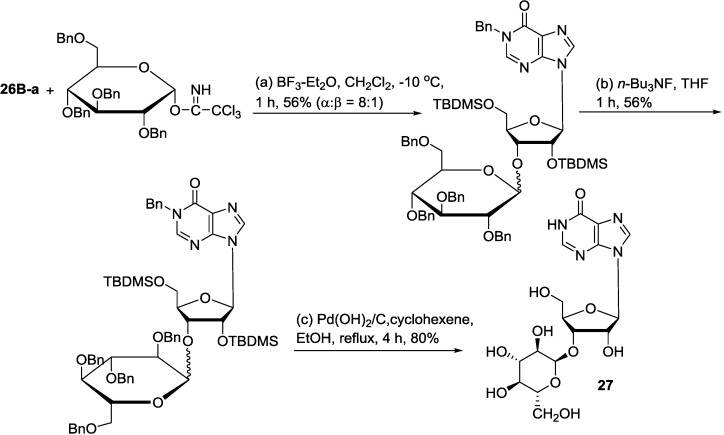
Synthesis of avinosol (**27**).

To further confirm the correct structure of the new inosine disaccharide, the four stereoisomers 2′-*O*-(α-d-glucosyl)- and 2′-*O*-(β-d-glucosyl)inosine and 3′-*O*-(α-d-glucosyl)- and 3′-*O*-(β-d-glucosyl) inosine were synthesized. The synthetic approach to these compounds must include an *O*-glycosylation step to form the disaccharide. Although there are plenty of examples of efficient nucleoside formation from simple, elaborated, or disaccharidal glycosyl donors, there are few cases of successful *O*-glycosylation of a nucleoside [63,99]. Attachment of a glucopyranosyl subunit at *O*-3′ of inosine would represent a rare example of *O*-glycosylation of a purine nucleoside because it requires overcoming the double obstacle of steric hindrance and competing depurination. Glycosyl acceptor **26B-a** was prepared through two steps (Scheme 2). The protection of inosine (**26**) with tert-butyldimethylsilyl chloride in pyridine gave a mixture of 2′,5′-di-*O*- and 3′,5′-di-*O*-(tert-butyldimethylsilyl) inosines (**26A-a**, **26A-b**). *N*-1-Benzylation of the **26A-a** and **26A-b** mixture with benzyl bromide yielded the glycosylation acceptor mixture of **26B-a** and **26B-b**, which was separated by Silica gel column chromatography. The TBDMS moiety is known to migrate to adjacent hydroxyl groups (from **26B-a** to **26B-b** and vice versa) under basic conditions [100]. Thus, the second step was performed without separation of **26A-a** and **26A-b**. Benzyl-protected glucopyranosyl donor was prepared according to a literature procedure [101]. The nonparticipating benzyloxy group at C-2″ was expected to direct α-glycosylation. Scheme 3 showed the synthetic procedure used for the synthesis of 3′-*O*-(α-d-glucosyl)inosine (**27**) [66].

**Scheme 4:**
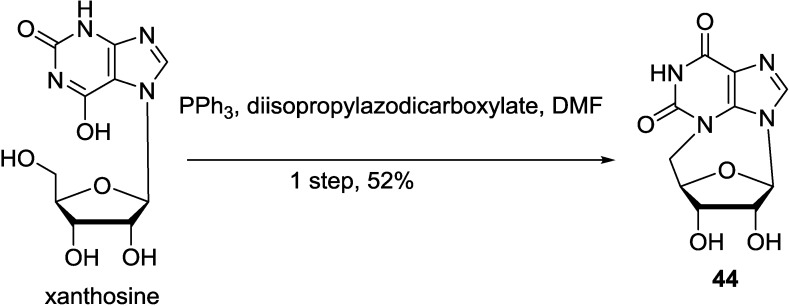
Synthesis of *N^3^*,5′-cycloxanthosine (**44**).

A reappraisal of the data for xanthosine suggested an alternative cyclonucleoside structure, namely, *N^3^*,5′-cycloxanthosine (**44**). First it was synthesized in 1963 [102], and further characterized by ORD studies [103], *N^3^*,5′-cycloxanthosine remained dormant in the scientific literature until 2004, at which time it came to Robert’s attention through the publication of a convenient one-step synthesis from xanthosine (Scheme 4) [79].

## 6. Biosynthetic Pathways

Nucleosides are vital components of all living cells and involved in several biological processes, the biological properties and the chemistry of some nucleosides have been well studied. Some of these molecules are even developed to be approved as drugs. However, the biosynthetic pathways of some promising molecules remain largely unexplored.

**Scheme 5:**
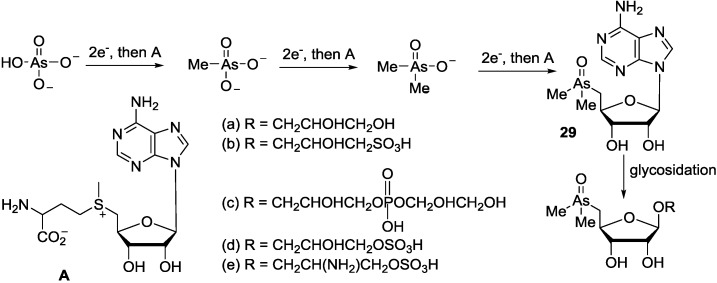
Proposed biosynthetic pathway for dimethylarsinylribosides.

Arsenic is naturally present in seawater at concentrations of 2–3 μg dm^−3^, mainly as arsenate. The major forms of arsenic in marine algae are dimethylarsinylribosides [104]. 5a-e, which are probably metabolized to arsenobetaine (Me_3_As^+^CH_2_CO_2_^−^), the usual form of arsenic in marine animals [105] within food chains. It has been proposed that algae biosynthesized dimethylarsinylribosides from absorbed oceanic arsenate by mechanisms first described by Challenger [106] for the biosynthesis of trimethylarsine by microorganisms, and involving *S*-adenosylmethionine (AdoMet) [107] as the methyl donor and 5′-deoxy-5′-dimethylarsinyladenosine (**29**) as a key intermediate (Scheme 5) [69].

**Scheme 6:**
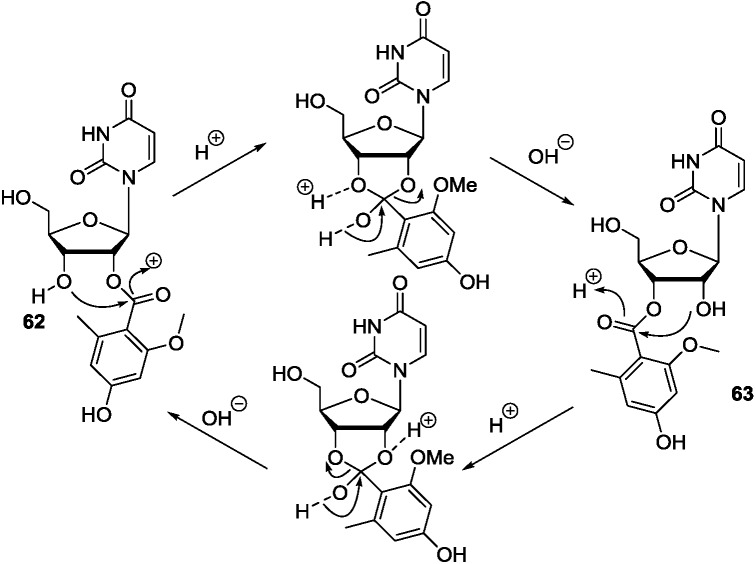
Mechanism of the interconversion for **62** and **63** in MeOH-H_2_O.

2′/3′-Transesterification has been known to occur in some ribose derivatives [108,109,110]. The interconversion between **62** and **63,** which were isolated from the fungus *A. versicolor*, was observed during the extraction and purification processes [93]. The 2′-*O*-acyl ribofuranoside or 3′-*O*-acyl ribofuranoside was labile and reactive in MeOH-H_2_O solution and underwent the intramolecular acyl migration to produce isomers. The aroyl in **62** was transferred from the 2′-OH position to the neighboring 3′-OH on the ribose moiety via an *ortho*-acid ester intermediate [111], yielding the regioisomer **63** (Scheme 6). This aroyl migration was reversible, which finally achieved a dynamic equilibrium (*n_1_*:*n_2_* = 7:10, approximately).

## 7. Conclusions

Up to June 2014, studies on marine nucleosides have been focused on the structure, bioactivity, synthesis and proposed biosynthetic pathway. Several bioactive marine nucleosides have been isolated from marine organisms. According to the reported data, marine sponges are the best source of these marine nucleosides. The heterocyclic moiety in marine nucleoside is either a substituted pyrimidine, purine or pyrrolo[2,3-d]pyrimidine moiety. The sugar moiety is either d-arabinose, d-ribose, 2′-deoxyribose, 2′,3′-didehydro, 2′,3′-dideoxyribose or a substituted xylose sugar. The discoveries of these remarkable biological activities found in the marine nucleosides have promoted the great amount of research work on the chemical synthesis of various analogs of these nucleosides. In some cases the compounds have been synthesized prior to the isolation from marine source. These molecules exhibited antiviral, anticancer, vasodilator, muscle relaxant, and hypertensive activities. Among them, the biological activity of the arabinosides is most prominent. Ara-A (**1**) is one of the best antiviral drugs. The searching for novel analogs of natural nucleosides with potential biological properties has driven the rapid progress in the area of nucleoside chemistry research. Marine nucleosides have provided new “Lead compounds” for drug design, particularly in the area of viral and parasitic infections. Several analogs of bioactive marine nucleosides have been synthesized and evaluated for biological activities. Consequently marine organisms can be a promising source for this class of bioactive compounds. Thus, much more investigations on the chemistry and the biological properties of marine nucleosides should be carried out in order to disclose the potency, selectivity, toxicity and availability of the bioactive marine nucleosides.
